# A genome-wide association and meta-analysis reveal regions associated with seed size in cowpea [*Vigna unguiculata* (L.) Walp]

**DOI:** 10.1007/s00122-019-03407-z

**Published:** 2019-07-31

**Authors:** Sassoum Lo, María Muñoz-Amatriaín, Samuel A. Hokin, Ndiaga Cisse, Philip A. Roberts, Andrew D. Farmer, Shizhong Xu, Timothy J. Close

**Affiliations:** 1 Department of Botany and Plant Sciences, University of California, Riverside, CA 92521 USA; 2 Department of Soil and Crop Sciences, Colorado State University, Fort Collins, CO 80523 USA; 3 National Center for Genome Resources, Santa Fe, NM 87505 USA; 4 Centre d’Etude Régional pour l’Amélioration de l’Adaptation à la Sècheresse, ISRA/CERAAS, Thies, Senegal; 5 Department of Nematology, University of California, Riverside, CA 92521 USA

## Abstract

**Key message:**

This paper combined GWAS, meta-analysis and sequence homology comparison with common bean to identify regions associated with seed size variation in domesticated cowpea.

**Abstract:**

Seed size is an important trait for yield and commercial value in dry-grain cowpea. Seed size varies widely among different cowpea accessions, and the genetic basis of such variation is not yet well understood. To better decipher the genetic basis of seed size, a genome-wide association study (GWAS) and meta-analysis were conducted on a panel of 368 cowpea diverse accessions from 51 countries. Four traits, including seed weight, length, width and density were evaluated across three locations. Using 51,128 single nucleotide polymorphisms covering the cowpea genome, 17 loci were identified for these traits. One locus was common to weight, width and length, suggesting pleiotropy. By integrating synteny-based analysis with common bean, six candidate genes (*Vigun05g036000*, *Vigun05g039600*, *Vigun05g204200*, *Vigun08g217000*, *Vigun11g187000*, and *Vigun11g191300*) which are implicated in multiple functional categories related to seed size such as endosperm development, embryo development, and cell elongation were identified. These results suggest that a combination of GWAS meta-analysis with synteny comparison in a related plant is an efficient approach to identify candidate gene (s) for complex traits in cowpea. The identified loci and candidate genes provide useful information for improving cowpea varieties and for molecular investigation of seed size.

**Electronic supplementary material:**

The online version of this article (10.1007/s00122-019-03407-z) contains supplementary material, which is available to authorized users.

## Introduction

Cowpea [*Vigna unguiculata* (L.) Walp] is a multipurpose crop and a major source of dietary protein, fiber, vitamins, and minerals for millions of people and livestock in sub-Saharan Africa. Eaten in the form of fresh seeds, dry seeds, and fresh pods and as forage, cowpea is also an important crop in some parts of Asia, Latin America, and the USA (Dadson et al. 2005; Singh 2014). Cowpea is also an additional source of income for smallholder farmers in Africa, and the impact on household economies depends largely on seed appearance including seed size.

Since the beginning of agriculture, increased seed size has been a main domestication target as an important component of grain yield. Domesticated crops produce larger seeds compared to their wild ancestors. Seed size has several agronomically important impacts. Lush and Wien (1980) reported that large seeded cowpea emerged earlier than small seeded types when planted deeply (up to 5 cm) and produce larger plants during early development. Cowpea seed size is an essential market trait in present-day Africa and other parts of the world. Consumers tend to prefer larger seeds (Mishili et al. 2009). Understanding the genetic basis of cowpea seed size is fundamental for breeding for this complex trait. Classical inheritance analysis suggested that at least eight loci control cowpea seed size (Drabo et al. 1984). Since the first publication of quantitative traits loci (QTLs) of seed weight by molecular markers (Fatokun et al. 1992), efforts have been made to identify QTLs associated with seed size based on seed mass using mapping populations with different genetic backgrounds (Andargie et al. 2014; Huynh et al. 2018; Lo et al. 2018; Lucas et al. 2013; Pan et al. 2017). All of these QTLs were discovered through linkage mapping using small populations. As a result, these QTLs are quite wide making the identification of causative genes challenging. Recently developed high-density genotyping tools and diverse germplasm subsets have made it possible to explore the genetic basis of this complex trait at finer resolution. To this aim, a genome-wide association study (GWAS) was conducted to investigate the genetic basis of seed size-related traits including seed length, width, and density using the recently developed genetic and genomic resources. Association mapping as described by Zhu et al. (2008) is a valuable tool to better understand the genetic basis of complex traits in plants. It has been widely adopted in several crop species to identify QTLs and to find candidate genes (Zhu et al. 2008). This approach enables the identification of genomic regions with finer resolution because of the smaller linkage disequilibrium in an association panel (Nordborg and Weigel 2008) as well as a larger population size.

A few GWA studies have been reported in cowpea for root architecture (Burridge et al. 2017), pod length (Xu et al. 2017), and black seed coat color (Herniter et al. 2018). No association mapping study has been reported on seed weight, length, width, and density in cowpea to date. Here, we conducted GWAS and meta-analysis to enhance our knowledge of the genetic architecture of seed size in cowpea. Meta-analysis (Rudner et al. 2002) is a useful tool through which GWAS results from different environments can be statistically pooled. This technique has been widely used in medical and social sciences. Seed size is known from studies on other plants to be controlled by various genes involved in different mechanisms including embryo and endosperm growth (Venglat et al. 2014). Genes affecting seed size have been cloned in *Arabidopsis* including *APETALA2* (*AP2*), *SHB1* and *IKU1* (Jofuku et al. 2005; Wang et al. 2010; Zhou et al. 2009). In soybean, *GmCYP78A10* and *GmCYP78A72* have been shown to play an important role in controlling seed size (Wang et al. 2015; Zhao et al. 2016). Here, we studied the genetic basis of seed size in domesticated cowpeas and identified single nucleotide polymorphisms (SNPs) significantly associated with seed size-related traits as well as promising candidate genes for which syntelogs in common bean (*Phaseolus vulgaris*) have been reported as seed weight candidate genes.

## Materials and methods

### Plant materials

A mini-core collection consisting of 368 accessions (Muñoz-Amatriaín et al. unpublished) was used for association mapping. The 368 accessions included landraces and breeding materials from 51 countries (Muñoz-Amatriaín et al. unpublished). It also included members of both the subspecies *unguiculata* and *sesquipedalis* (yardlong bean). The 368 lines were classified into six subgroups based on population structure (Muñoz-Amatriaín et al. unpublished). All accessions were grown under favorable conditions in the greenhouse and field in 2016 and 2017. Greenhouse growth was carried out at the University of California Riverside campus (33.57°N; 117.20°W) as follows: Seeds from each accession were grown in 3.8 L pots filled with UCmix3 with temperatures at 35 °C day and 23 °C night. Mature, dried pods were harvested from each plant. For field experiments, each accession was planted in a single-row plot at the UCR Citrus Experiment Station, California, USA (UCR-CES, 33.97°N, 117.34°W; Field11) and at the Coachella Valley Agricultural Research Station, California, USA (CVARS, 33.52°N, 116.15°W). In field trials, mature, dried pods were harvested randomly as to minimize bias.

### Phenotypic evaluation and statistical analysis

Seed size-related traits were evaluated as 100-seed weight (g), seed length (mm), seed width (mm), and seed density (g/cm^3^) in greenhouse and field experiments. The phenotyping for seed length and width was based on the average length and width of 10 seeds of each accession measured using a digital caliper. Seed density was calculated for each accession based on seed mass and its volume following the formula:

$${\text{seed}}\;{\text{density}} = \frac{{{\text{seed}}\;{\text{mass}}}}{{\frac{\pi }{6}*{\text{length}}*{\text{width}}^{2} }}$$

Among the seed size traits, seed weight was measured in three environments. Broad sense heritability was estimated based on the data across the three environments. The variance components were estimated with the PROC VARCOMP procedure in SAS, and broad sense heritability calculated as follows:

$$H^{2} = \frac{{V_{\text{G}} }}{{V_{\text{G}} + V_{\text{E}} }}$$

where *V*_G_ is the variance of genotype and *V*_E_ is the variance of the experimental error.

Pearson’s correlation coefficients were calculated using the cor.test () function in R (Ihaka and Gentleman 1996). The correlation coefficient was calculated between seed mass, seed length, seed width, and seed density.

### SNP genotyping

Total genomic DNA was extracted from dried leaves collected from one plant of each accession using Plant DNeasy (Qiagen, Germany). Total DNA was quantified using a Quant-IT dsDNA Assay Kit (Thermo Fisher Scientific, USA). The 368 accessions were genotyped using the Cowpea iSelect Consortium Array containing 51,128 SNPs (Muñoz-Amatriaín et al. 2017). Genotyping was conducted at the University of Southern California Molecular Genomics Core facility (Los Angeles, California, USA). SNPs were called using GenomeStudio software V.2011.1 (Illumina, Inc. San Diego, CA). For GWAS, data were filtered by removing SNPs with more than 25% missing calls, and minor allele frequency (MAF) less than 0.05. The physical positions of these SNPs were determined using the IT97 K-499-35 reference genome (Lonardi et al. 2019).

### Genome-wide association study and meta-analysis

GWAS was conducted on 100-seed weight (measured in three locations), length, width, and density (each measured in one location; CVARS). GWAS was performed using the mixed linear model (Zhang et al. 2010) in TASSEL V5.0 (http://www.maizegenetics.net/tassel). Principal component analysis (PCA) and a kinship coefficient matrix (*K*) were generated in TASSEL. PCA was used to account for population structure (*Q*), and *K* was used to correct for relatedness of accessions. The percentage contribution of each SNP to the total phenotypic variation was calculated using marker R^2^ values (computed by TASSEL) multiplied by 100.

Seed weight was measured in three environments. GWAS was first performed separately for each environment, and then results were combined via a meta-analysis. A simple meta-analysis procedure recommended by Kang et al. (2014) was used to increase power of association and detect G × E interaction loci. This method essentially treats multiple environments as multiple populations. Loci that have different effects across different environments are G × E interaction loci. The method has been used in detecting interaction loci in a multiple environment mouse experiment (Kang et al. 2014). Among several specialized algorithms, Fisher’s method was adopted because the method only requires the *p* values across multiple environments. However, this method requires a comprehensive tool to prove the distribution. Therefore, we developed a similar method that does not need the proof of the distribution. This method is called the Probit method. Let $p_{k}$ be the *p* value from environment *k* for $k = 1, \ldots ,m$, where m is the number of environments. The test statistic is defined as

$$X = \sum\limits_{k = 1}^{m} {\left[ {\varPhi^{ - 1} \left( {1 - 0.5p_{k} } \right)} \right]^{2} }$$

where $\varPhi^{ - 1} ()$ is called the probit function, which is the inverse function of the standardized normal distribution. In other words, if $p = \varPhi (z)$ and $p$ is a standardized uniform variable, then $z = \varPhi^{ - 1} (p)$ is a standardized normal variable. The square of a standardized normal variable is a Chi-square variable with one degree of freedom. The sum of m one-degree Chi-square variables is a Chi-square variable with m degrees of freedom. Under the null model ($p_{k}$ is a standardized uniform variable between 0 and 1), this test statistic follows a Chi-square distribution with $m$ degrees of freedom (no proof is needed). The new *p* value was calculated as follows:

$$P_{\text{probit}} = 1 - \Pr \left( {X_{m}^{2} \le X^{2} } \right)$$

The result is much the same as in Fisher’s method; the only difference is that the Chi-square distribution is given without the need for proof. The threshold for genome-wide significance cutoff was applied based on Bonferroni correction at *α* = 0.05.

### Candidate genes and syntelogs

The regions significantly associated with the traits were localized on the cowpea reference genome (Lonardi et al. 2019) to determine the underlying candidate genes. The significant regions were also compared to syntenic regions on the common bean genome (Schmutz et al. 2014) using the legumeinfo.org instance of the Genome Context Viewer (GCV) (Cleary and Farmer 2017) to determine a list of common bean genes (syntelogs). Synteny relationships between genes were based on their assignment to the same gene families, using hmmsearch at a threshold of 1e^−10^ against Hidden Markov Models representing legume gene families https://legumeinfo.org/data/public/Gene_families/legume.genefam.fam1.M65K; we also required them to occur in regions of similar genic content (10 or more homologous genes with conserved order in a neighborhood of 20 genes surrounding the candidate). Using functions of cowpeamine and legumemine (https://mines.legumeinfo.org), syntelogs were further intersected with a list of genes associated with seed weight as reported in Schmutz et al. (2014) based on their homology to functionally characterized Arabidopsis genes. Cowpea genes homologous to the common bean genes present in the intersection were considered to be strong candidate genes associated with seed weight.

## Results

### Phenotypic variation and seed size trait correlations

**Fig. 1:**
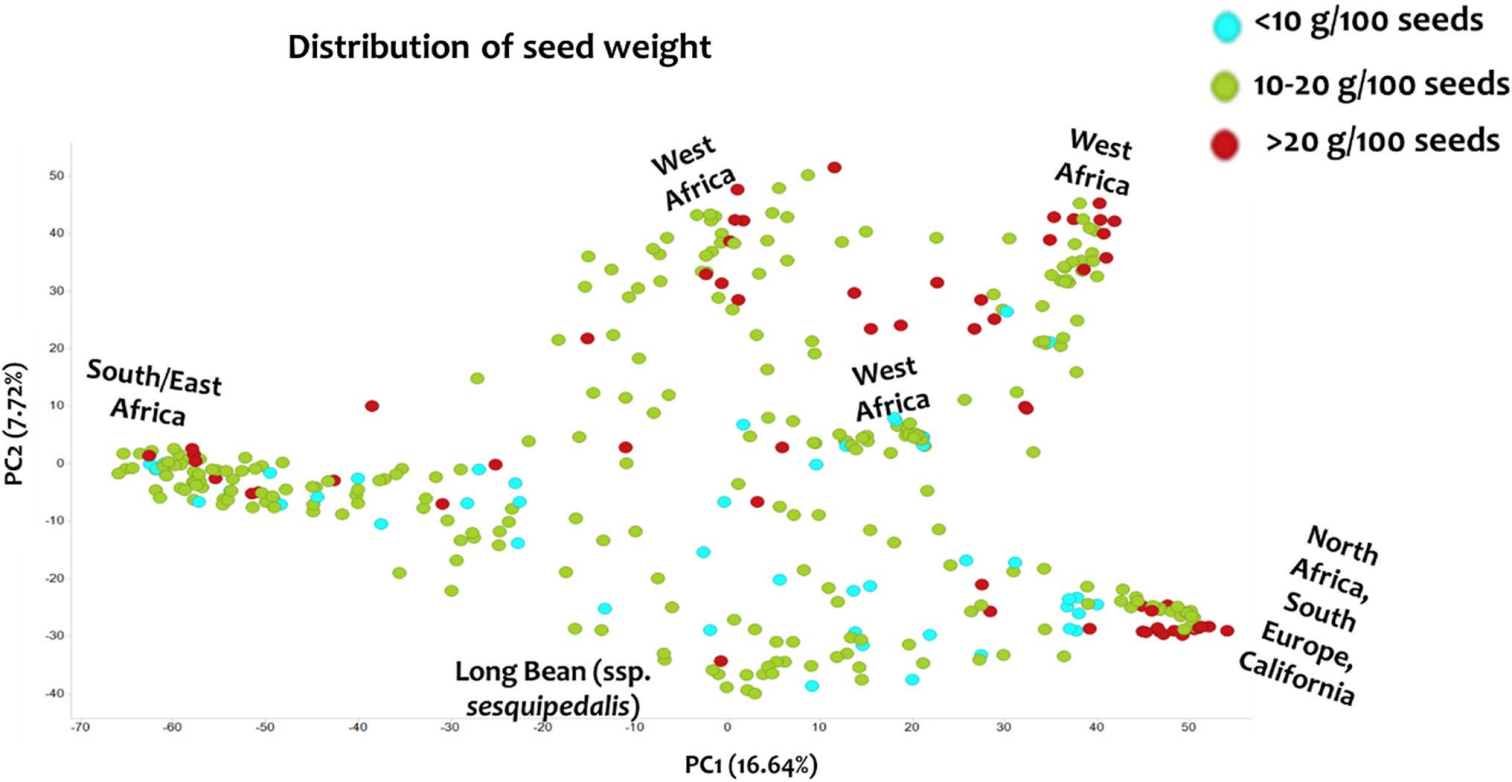
Principal component analysis of the 368 accessions colored by seed weight range

The phenotyping panel contained 368 cowpea accessions comprising both landraces and breeding materials, classified into six subgroups based on population structure (Muñoz-Amatriaín et al. unpublished). Seed size-related traits were evaluated based on seed weight, length, width and density. Mean and standard deviation were determined for each trait (Table 1). Broad sense heritability of seed weight was calculated, and the estimated value of 0.61 suggests relatively high heritability. The 368 accessions show a variation of grain sizes ranging from 5 to 31 g per 100 seeds. Principal component analysis (PCA) showed the distribution of “seed weight” (Fig. 1) in the diversity panel. The frequency distribution of seed weight is also shown in Supplemental Figure 1. To see the relationship between seed size traits, Pearson’s correlation coefficients were calculated between seed mass, length, width, and density. Seed mass is highly correlated (positive) with seed length (0.84) and width (0.89). Furthermore, seed width and length showed moderate correlation (0.67), possibly because of the yardlong bean accessions in the diversity set. Negative correlations were observed between density and length (− 0.67), and density and width (− 0.85) indicating that as seeds are larger they are also less dense.

**Table 1:** Mean and standard deviation of the seed size traits in the panel

Trait	Location	Mean	Standard deviation
Seed weight (g)	GH	15.22	4.85
	UCR-CES	17.49	5.34
	CVARS	14.35	4.75
Seed length (mm)	UCR-CES	6.59	1.36
Seed width (mm)	UCR-CES	4.41	0.91
Seed density (g/cm^3^)	UCR-CES	2.27	0.83

### Association mapping

**Fig. 2:**
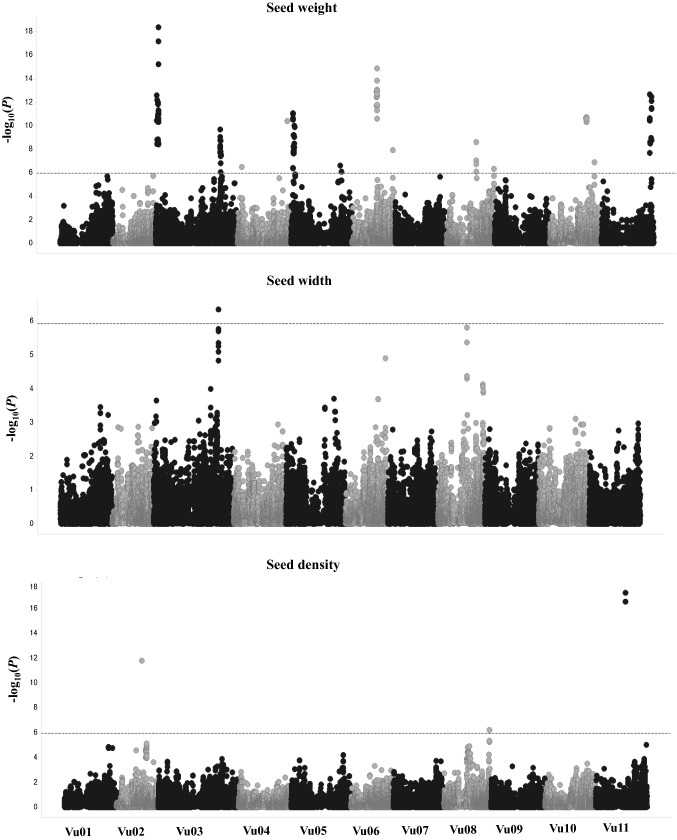
Manhattan plot of GWAS meta-analysis for seed weight and GWAS for seed width and density. Negative Log_10_*p* values are plotted against physical position on each of the 11 chromosomes. Dashed line indicates Bonferroni-corrected genome-wide significance threshold

A total of 42,711 polymorphic SNPs were used for GWAS. A total of 17 loci were identified for the seed size-related traits (Fig. 2; Tables 2, 3). A hot spot was identified on chromosome Vu03, where QTLs for seed weight (Sw3.2), width (Swi3) and length coincided. Colocalization of seed size-related trait QTLs suggests pleiotropy or physical linkage of genes controlling seed size in cowpea.

**Table 2:** List of significant QTLs for seed weight from GWAS meta-analysis results

QTL	Peak SNP	Chr.	Position	− Log_10_*p*	Comment
Sw3.1	2_26016	3	2223359	18.36	GH, UCR-CES, CVARS, Meta
Sw3.2	2_40922	3	51562014	9.67	UCR-CES, Meta
Sw4.1	2_14347	4	3483218	6.51	Meta
Sw4.2	2_25894	4	39757606	10.41	CVARS, Meta
Sw5.1	2_07617	5	1783613	11.08	UCR-CES, CVARS, Meta
Sw5.2	2_08022	5	39230104	6.63	UCR-CES, Meta
Sw6.1	2_07078	6	19698161	14.84	GH, UCR-CES, CVARS, Meta
Sw6.2	2_07358	6	32476947	7.92	Meta
Sw8.1	2_50483	8	23709814	8.61	Meta
Sw8.2	2_17605	8	37710852	6.32	Meta
Sw10.1	2_50509	10	29351777	10.73	CVARS, Meta
Sw10.2	2_08400	10	35932948	6.87	UCR-CES, Meta
Sw11	2_28551	11	38568662	12.69	GH, UCR-CES, CVARS, Meta

**Table 3:** List of significant QTLs for seed width and density from GWAS results

Trait	QTL	Peak SNP	Chr.	Position	− Log_10_*p*	*R*^2^ (%)	Alleles	Effect
Seed width	Swi3	2_29499	3	52931236	6.35	7.09	A/C	− 0.59
Seed density	Sd2	2_09625	2	20857395	11.82	14.84	G/T	0.06
	Sd8	2_27144	8	36984438	6.22	7.06	A/C	− 0.63
	Sd11	2_41753	11	24148832	17.27	21.93	C/T	0.12

#### Seed weight

GWAS was performed separately for the three environments, and results were combined via the Probit meta-analysis (see “Materials and methods” section). The meta-analysis identified 13 significant regions on chromosomes 3, 4, 5, 6, 8, 10, and 11 (Fig. 2; Table 2; Supplemental Table 1). Those loci included all the loci identified in each single environment (Supplemental Table 1), plus four loci that were not detected in any single environment. Four loci were consistent across two environments (UCR-CES and CVARS), while three loci were common to all three environments (GH, UCR-CES and CVARS), suggesting a presence of a significant G × E interaction. Effects and *R*^2^ values of significant SNPs were calculated for each single environment and are reported in Supplemental Table 1. We also used Fisher’s meta-analysis to combine results of the three replications for seed weight. The results are almost indistinguishable from the Probit method developed here (see “Materials and methods” section). The results of both methods are shown in Supplemental Table 2. Recall that four additional loci were detected by the meta-analysis. The effects of the four loci may not be sufficiently large to be detected in any single environment, but when results from individual environments were pooled via the meta-analysis, they were significant.

#### Seed length, width, and density

One significant region was associated with seed width on Vu03, and three regions were associated with seed density on Vu02, Vu08, and Vu11 (Fig. 2; Table 3; Supplemental Table 1). The strongest QTL (Sd11) was localized on Vu11 and explained the highest genetic variance (21.51%; Table 3; Supplemental Table 1). Significant loci for seed length were not identified, although three loci were detected slightly below the significance threshold. Since the Bonferroni-corrected threshold is very conservative, loci with levels slightly lower than the significance threshold are listed in Supplemental Table 1. Of the loci considered for seed length, one was common with the locus detected for seed weight (Sw3.2) and seed width (Swi3) on Vu03, and another one was common with the locus detected for seed density (Sd8) on Vu08.

### Candidate genes and syntelogs

All identified loci were aligned to the cowpea reference genome (Lonardi et al. 2019), and the underlying genes are listed in Supplemental Table 3. Synteny-based analysis performed with common bean using GCV (https://legumeinfo.org/lis_context_viewer) identified six syntelogs from the intersection with seed weight candidate genes reported by Schmutz et al. (2014). The six syntelogs along with their corresponding cowpea genes are given in Supplemental Table 4. The corresponding cowpea genes of the syntelogs include two for Sw5.1 (*Vigun05g036000*, *Vigun05g039600*), one for Sw5.2 (*Vigun05g204200*), one for Sw8.2 (*Vigun08g217000*), and two for Sw11 (*Vigun11g187000*, *Vigun11g191300*). The gene *Vigun05g036000* is annotated as cell wall protein while *Vigun05g039600* encodes a phosphate transporter PHO1. *Vigun05g204200* is annotated as encoding a polycomb group protein FERTILIZATION-INDEPENDENT ENDOSPERM and *Vigun08g217000* codes for a histidine kinase 2. The genes *Vigun11g187000* and *Vigun11g191300* are annotated as WD repeat-containing protein 61-like isoform 1 and delta (24)-sterol reductase-like protein, respectively. As comparative genomics is an efficient approach, we draw attention to these six interesting candidate genes.

## Discussion

Seed size is one of the key yield determinants. Understanding the underlying genetic factors of cowpea seed size can help breeders develop improved varieties with a range of seed sizes. Also, identifying genes that could control seed size variation in domesticated cowpea can provide important insights into the genetic basis of adaptation, as seed size has been recognized as an important contributor to adaptation in plants (Chapin III et al. 1993; Igea et al. 2017). Previous studies using mapping populations from different genetic backgrounds have identified QTLs for seed size based on seed mass (Andargie et al. 2014; Fatokun et al. 1992; Huynh et al. 2018; Lo et al. 2018; Lucas et al. 2013; Pan et al. 2017). However, these studies have provided limited information on potential candidate genes for seed size. In cowpea, determining the genetic basis of complex traits has become increasingly effective due to the availability of the genome sequence (Lonardi et al. 2019) and high-density genotyping (Muñoz-Amatriaín et al. 2017). As the next step to gain new knowledge on the genetic control of seed size, we used GWAS and meta-analysis to identify loci and candidate genes for seed size-related traits at a higher resolution that has been possible previously.

The meta-analysis identified 13 loci for seed weight (Fig. 2; Table 2) including four loci that were not detected in any single location (Table 2). The power of meta-analysis to identify additional loci in the combined data set that were not detected in the single environment due to insufficient power provides support for its utility. As has been shown in many studies in medical and social sciences, meta-analysis can overcome the limits of an individual environment by increasing the resolution power and reducing false-positive findings (Evangelou and Ioannidis 2013). It is well known that increasing sample size can increase statistical power and decrease Type 1 error (Wang and Xu 2019). Meta-analysis is much like combining multiple populations (environments) into a pooled data and thus serves to increase the sample size. Rather than pooling the original data, this meta-analysis pools the results (the *p* values). This result also revealed a presence of a significant G × E interaction with some loci being location specific. The presence of G × E interaction loci noted here confirms the complexity of seed weight. Overlap between our meta-analysis result and previous studies was found. BLAST searches of RLFP probe sequences from Fatokun et al. (1992) against the reference genome sequence (Lonardi et al. 2019) revealed that the two QTLs identified by Fatokun et al. (1992) seem to correspond to Sw3.2 and Sw6.1. Furthermore, Fatokun et al. (1992) reported that one of the QTLs (the one corresponding to Sw3.2 here) is orthologous to a seed weight QTL in mung bean (*Vigna radiata*), suggesting that the genomic region has remained conserved through evolution. Also, Pan et al. (2017) reported a locus for grain weight which overlapped with Sw3.2. The location of QTL Sw8.2 on Vu08 overlapped with that identified from several independent studies (Huynh et al. 2018; Lo et al. 2018; Lucas et al. 2013). However, Sw6.2 on Vu06 is very close to a locus identified by Lo et al. (2018) for seed weight. This study may serve to refine this locus, or may indicate a novel locus. Sw3.1, Sw4.1, Sw4.2, Sw5.1, Sw5.2, Sw8.1, Sw10.1, Sw10.2, and Sw11 represent novel associated loci, further suggesting the complexity of seed size. Consistent with results from (Drabo et al. 1984), our study demonstrated that at least eight loci control cowpea seed size. Our meta-analysis result can guide the choice of QTL targeted for marker-assisted selection.

In this study, we also identified significant regions for seed width and density. The locus for seed width (Swi3) was in the same region as Sw3.2. Also, a clear peak for seed length was noted on the same genomic region. We note also that this region overlapped with a region identified for grain weight by Pan et al. (2017). Taken together with the high correlation between seed mass, width, and length, we hypothesize that this QTL has a pleiotropic effect. However, the possibility of coexistence of multiple genes should not be excluded due to the complexity of these traits. Additional studies are necessary to further explore any of these hypotheses. In addition to their contribution to yield, seed width, density, and length are important traits that have impact on the market value of cowpea. These QTLs will be a valuable resource for the improvement of cowpea.

We identified genes within these QTLs regions (Supplemental Table 3) using the annotated cowpea reference genome (Lonardi et al. 2019). Among the associated loci for seed weight, six particular candidate genes were identified based on comparative genomic analysis with common bean. Two interesting candidate genes for Sw5.1 were *Vigun05g036000* and *Vigun05g039600*. The former encodes a cell wall protein, which was reportedly associated with seed size (Cheng et al. 1996; Jin et al. 2009; Weber et al. 1996). The latter, *Vigun05g039600*, encodes a phosphate transporter PHO1. In Arabidopsis, a PHO1 gene has been reported to be a positive regulator of seed development that affects both cell size and cell number (Zhou et al. 2009). Since several genetic pathways are known to control seed size, this gene is a promising candidate. Synteny-based analysis suggested that *Vigun05g204200*, which is a potential candidate for Sw5.2, is annotated as encoding a polycomb group protein FERTILIZATION-INDEPENDENT ENDOSPERM (FIE). FIE genes are involved in endosperm development (Ohad et al. 1996) and have been shown to regulate seed size (Folsom et al. 2014). The candidate gene for Sw8.2 was *Vigun08g217000* which codes for a histidine kinase 2. Interestingly, *Vigun08g217000* has been identified as potential candidate gene for increased organ size during cowpea domestication (Lonardi et al. 2019) and its Arabidopsis ortholog *AHK2* has been shown to regulate seed size (Bartrina et al. 2017; Riefler et al. 2006). Similarly, two candidate genes for Sw11 were determined: *Vigun11g187000* and *Vigun11g191300*. The former is annotated as WD repeat-containing protein 61-like isoform 1. WD repeat proteins are mainly involved in cellular processes including cell division (van Nocker and Ludwig 2003). In addition, the Arabidopsis gene *AT2G34260*, which encodes a WD repeat protein, is required for embryo and endosperm development (Bjerkan et al. 2012). *Vigun11g191300* encodes a delta (24)-sterol reductase protein and is an ortholog of the Arabidopsis *DIMINUTO* gene which has been shown to regulate cell elongation (Takahashi et al. 1995). *Vigun11g191300* is a strong candidate as seed size is influenced by multiple pathways.

In summary, combined GWAS meta-analysis and comparative genomics have led to a better understanding of the genetic basis of seed size-related traits. QTLs harboring candidate genes have been identified which deserve further in-depth studies to explore their possible roles in cowpea seed size. A direct result of the present study has been to establish genetic markers of these variants, which now are available to facilitate cowpea breeding for improved varieties with a range of seed sizes.

### Author contribution statement

SL, MMA, SAH, SX, and TJC designed the study. SL analyzed the data with help from MMA, SAH, ADF, and SX. SL wrote the paper with inputs from SX, SAH, and TJC. MMA, TJC, PAR, NC read and reviewed the paper.

## Electronic supplementary material

Below is the link to the electronic supplementary material.

Supplementary material 1 (XLSX 28 kb)

Frequency distribution of seed weight in the diversity panel (PPTX 60 kb)

Supplementary material 3 (XLSX 36 kb)

Supplementary material 4 (XLSX 2250 kb)

Supplementary material 5 (XLSX 151 kb)

Supplementary material 6 (XLSX 10 kb)
