# Supplementary material for: A genome-wide association and meta-analysis reveal regions associated with seed size in cowpea [Vigna unguiculata (L.) Walp]
Source: Theor Appl Genet. 2019 Jul 31;132(11):3079–87. doi: 10.1007/s00122-019-03407-z (PMC6791911; doi:10.1007/s00122-019-03407-z)
Supplement: Supplementary file 2 — Frequency distribution of seed weight in the diversity panel (PPTX 60 kb) [file 122_2019_3407_MOESM2_ESM.pptx]

## Slide 1
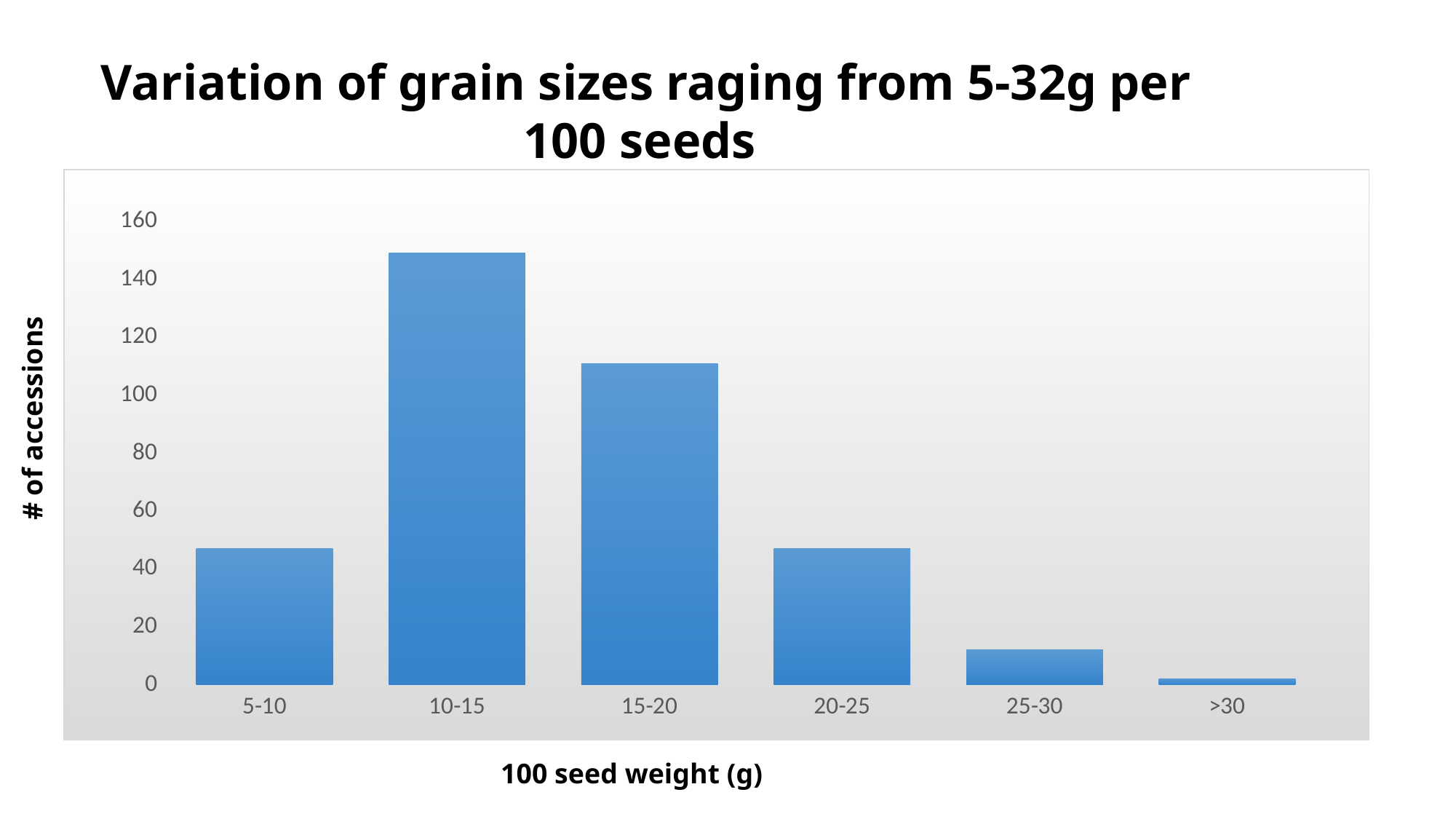

Variation of grain sizes raging from 5-32g per 100 seeds
### Chart
| Category | # accessions |
|---|---|
| 5-10 | 47.0 |
| 10-15 | 149.0 |
| 15-20 | 111.0 |
| 20-25 | 47.0 |
| 25-30 | 12.0 |
| >30 | 2.0 |# of accessions
100 seed weight (g)
